# Office-based simple frailty score and central blood pressure predict mild cognitive impairment in an apparently healthy Japanese population: J-SHIPP study

**DOI:** 10.1038/srep46419

**Published:** 2017-04-13

**Authors:** Maya Ohara, Katsuhiko Kohara, Yoko Okada, Masayuki Ochi, Tokihisa Nagai, Yasumasa Ohyagi, Yasuharu Tabara, Michiya Igase

**Affiliations:** 1 Department of Geriatric Medicine and Neurology, Ehime University Graduate School of Medicine, Ehime, Japan; 2 Faculty of Collaborative Regional Innovation, Ehime University, Ehime Japan; 3 Center for Genomic Medicine, Kyoto University Graduate School of Medicine, Kyoto, Japan

## Abstract

Frailty is associated with cognitive impairment and can be used to identify people at high risk for dementia. We developed a simple frailty (SF) score using a combination of low hand grip strength (<32.5 kg in men, <19.5 kg in women), and short one-leg standing time (<20 seconds). These can be easily measured in the clinician’s office when seeing patients. We investigated the possible association between SF score and mild cognitive impairment (MCI) in a cross-sectional study with 838 independent middle-aged to elderly participants (319 men, mean age 65.1years). In total, 118 participants were diagnosed with MCI. A SF score of 2 was significantly associated with the presence of MCI (odds ratio 4.6, 95% confidence interval: 1.9–6.9, p = 0.0001) even after adjustment for age and sex. Stepwise regression analyses showed that a SF score of 2 was associated with the presence of MCI, independently of central pulse pressure and silent cerebral infarcts. These findings indicate that the SF score is a useful frailty parameter to predict MCI in an apparently independent population.

Identification of people at high risk for dementia is a corner-stone, early intervention strategy to prevent progression to dementia. Mild cognitive impairment (MCI), especially amnestic MCI, is a prodrome of dementia (including Alzheimer’s disease) and has been researched as a possible target for early intervention^1^. Recent consensus from the International Association of Gerontology and Geriatrics stresses the importance of case-findings by physicians and health professionals^2^.

Frailty is a state of increased vulnerability owing to poor resolution of homoeostasis after a stressor event that increases the risk of adverse outcomes, including disability^3^. A close association between frailty and dementia has been demonstrated^4^. Criteria proposed by Fried *et al*.^5^ are most frequently used in clinical practice, and comprise three or more of five conditions: slowness, weakness, shrinking, inactivity and exhaustion. Slow walking speed, a component of frailty, has also been significantly associated with MCI^6^ and future development of MCI^7^. Subjective cognitive decline in combination with slow gait speed has been defined as motoric cognitive risk syndrome^8^, which was reported to be a risk factor for future development of dementia^9^.

Recently, we developed a simple frailty (SF) score using the combination of low hand grip strength and short one-leg standing (OLS) time, both of which can be easily measured in the clinician’s office^10^. SF score was significantly associated with systemic frailty, including cognitive function^10^. In addition, SF score was significantly associated with advanced end organ damage including brain small vessel disease (SVD)^10^.

Based on these findings, we hypothesised that SF score could predict the presence of MCI in an apparently healthy and independent population, and may be a useful tool to identify people at high risk. We investigated the possible association between SF score and MCI in a cross-sectional study. We also evaluated SF strict score based on the Asian definition of sarcopenia^11^. Further, we evaluated pulmonary function, arterial stiffness, central blood pressure (BP) and SVD as possible mechanisms underlying the association between SF score and MCI.

## Methods

### Study participants

The study population comprised 864 consecutive middle-aged to elderly participants who attended the medical check-up programme at the Ehime University Hospital between March 2006 and December 2014. Of the 864 participants who completed MCI screening, we analysed data for 838 who agreed with the study aims and protocols and had no history of symptomatic cardiovascular events (including stroke, transient ischaemic attack, coronary heart disease or congestive heart failure). Written informed consent for all procedures was obtained from all participants. This cross-sectional investigation was conducted as part of the Shimanami Health Promoting Program (J-SHIPP study), a longitudinal study evaluating factors related to cardiovascular disease, dementia and death^10^. The J-SHIPP series of studies was approved by the Ethics Committee of the Ehime University Graduate School of Medicine. All studies were performed in accordance with relevant guidelines and regulations.

### SF score

Participants were scored 1 point if their OLS time was <20 s, and 1 point if their hand grip strength was <32.5 kg (men) or <19.5 kg (women); these scores were summed to give the SF score^10^. We also analysed SF score using the Asian definition of sarcopenia (grip strength <26 kg in men or <18 kg in women), defined as participants’ SF strict score^11^.

Further we also evaluated the prediction of MCI using a definition of low grip strength (men <39 kg and women <20.3 kg) and low OLS time (<35.2 s) from the ROC curve for each parameter.

#### Hand grip strength

Hand grip strength was measured using a digital hand dynamometer (T.K.K. 5410; Takei Scientific Instruments Co. Ltd., Niigata, Japan), performed once on each side; the highest value was used for analysis^12^. The dynamometer’s minimum detectable hand grip strength was 0.1 kg. All participants who performed hand grip strength measurements were free from any condition (such as arthralgia) that might interfere with measurement. The cut-off value for the SF score in our study population was based on the bottom 20% in each sex. The intra-measurement reproducibility coefficient of variation (CV) was 5.0 ± 3.2% and the inter-measurement CV was 5.2 ± 3.7%^12^.

#### OLS test

The OLS test was performed with eyes open and measured using the leg selected by the participant. The time interval until the raised leg was put down was measured twice, with a maximum time of 60 s allowed. The better of the two recordings was used for statistical analysis. The cut off value for OLS time (less than 20 s) was based on previous findings^13^.

### Assessment of MCI

MCI was assessed using the Japanese version of the MCI screening method (MCI Screen), a 10-minute, computationally scored, staff-administered test^14^. The methodology for scoring the MCI Screen was developed by the Medical Care Corporation (Irvine, CA, USA). The MCI Screen is a brief neuropsychological test derived from the protocol of the Consortium to Establish a Registry for Alzheimer’s Disease 10-word recall test. The MCI Screen comprises three immediate recall tasks, a triadic comparison task, a judgment task, a delayed free recall task, a cued-recall task and a rehearsed recall task. MCI Screen results were submitted to the online analysis centre, and points scored by each screening test participant were used to diagnose MCI. Validity and specificity in the differentiation of normal aging from MCI and mild dementia have been described elsewhere, and the overall accuracy in discriminating both amnestic and mixed cognitive domain types of MCI from normal aging is 97%^14^. The MCI Screen has been translated into Japanese, and cross-validation has been confirmed using the Clinical Dementia Rating Scale as a reference (overall accuracy: 96.4%)^15^.

### Pulse wave velocity (PWV)

PWV was measured using a volume-plethysmograph (PWV/ABI; Omron Healthcare Co. Ltd., Japan). A detailed description of this device and the validity and reproducibility of its measurements have been published elsewhere^16^. Brachial-to-ankle PWV (baPWV) was calculated using the time interval between the wave fronts of the brachial and ankle waveforms (∆Tba) and the path length from the brachium to the ankle. Path lengths from the suprasternal notch to the brachium (Lb) or ankle (La) were calculated using the formulae: Lb = 0.2195 × height + 2.0734; La = 0.8129 × height + 12.328. Then, baPWV was calculated using the equation (La−Lb)/∆Tba.

### Radial waveform analysis and blood pressure (BP) measurement

The left radial artery pulse waveform was measured using an automated tonometric method (HEM-9000AI; Omron Healthcare Co. Ltd.) with participants in a sitting position after at least 5 min of rest. Brachial BP was measured simultaneously in the right brachium with an oscillometric device incorporated in the HEM-9000AI. The HEM-9000AI device is programmed to automatically adjust the pressure against the radial artery to obtain the optimal arterial waveform. Late systolic second peak BP (SBP2) was calculated by calibration with brachial systolic BP (SBP). Pulse pressure (PP) was calculated as PP = SBP − diastolic BP (DBP), and PP2 was calculated as SBP2 − DBP. The radial augmentation index (AI) was calculated as PP2/PP × 100(%)^17^. All measurements were repeated twice and the mean values were used for subsequent analyses. Radial AI and PP2 have been shown to accurately reflect transfer function-derived aortic AI and aortic PP, and were used as central BP-related values^17^.

### Assessment of silent brain lesions by magnetic resonance imaging (MRI)

In 733 participants, silent cerebral infarcts and white matter hyperintensities (WMHs), including periventricular hyperintensity (PVH) and deep subcortical white matter hyperintensity (DSWMH), were evaluated based on brain MRI findings using a 3-tesla scanner (Signa Excite 3.0 T; GE Healthcare, Milwaukee, WI, USA). Detailed methods have been previously reported^13^. In brief, silent lacunar infarction (SLI) was defined as areas of low signal intensity (3–15 mm diameter) on T1-weighted images, and areas of high intensity on T2-weighted and fluid-attenuated inversion recovery (FLAIR) images. Hyperintensities depicted on T2-weighted and FLAIR images in contact with the ventricular wall and located in the subcortical region were defined as PVH and DSWMH, respectively. PVH was further classified in five grades based on Japanese guidelines^13^: grade 0 = absent or only a ‘rim’; grade 1 = limited lesion-like ‘caps’; grade 2 = irregular ‘halo’; grade 3 = irregular margins and extension into the deep white matter; and grade 4 = extension into the deep white matter and subcortical portion. DSWMH was also classified in five grades^13^: grade 0 = absent; grade 1 = ≤3 mm small foci and regular margins; grade 2 = ≥3 mm large foci; grade 3 = diffusely confluent; and grade 4 = extensive changes in the white matter. The presence of WMHs was defined as PVH grade ≥2 and/or DSWMH grade ≥3. Images were analysed by two neurologists without clinical information about the participants. Images were analysed using OsiriX software (http://www.osirixviewer.com/).

### Postural instability

Postural instability was measured with a posturograph (Gravicorder G-5500; Anima Inc., Tokyo, Japan). This consists of an equilateral triangular footplate with three built-in vertical force transducers that determine instantaneous fluctuations in the centre of pressure^10^. Signals were processed by a direct-coupled amplifier and low-pass filters (cut-off frequency 10 Hz) and stored in a computer after analogue–digital conversion at a sampling rate of 20 Hz. Participants were instructed to maintain a static upright posture on the footplate with their feet together and watch a circular achromatic target placed 2 m in front of their eyes. Data were acquired for 1 min, beginning after the participant’s posture had stabilised. All measurements were performed barefoot with both arms held at the side of the body. Path lengths of the centre of pressure movement were considered parameters for movement of centre of gravity, and used as indices of postural stability^10^. Postural instability was defined as a path-length of centre of gravity of 86.92 cm or more by a ROC curve for the presence of MCI.

### Pulmonary function

Pulmonary function was measured using a spirometer (Microspiro HI-801; CHEST M.I., Inc., Tokyo, Japan) in 826 participants. Vital capacity (VC), forced vital capacity (FVC), and forced expiratory volume in 1 s (FEV1.0) were measured, and %VC and FEV1.0% were calculated^10^.

### Risk factor evaluation

Participants’ lifestyles, medical histories, and use of prescribed drugs were assessed with a questionnaire. All anthropometric measurements were performed by a trained nurse. Hypertension was defined as (any or all) SBP ≥ 140 mmHg, DBP ≥ 90 mmHg or use of an antihypertensive drug. Type 2 diabetes was defined as (any or all) fasting plasma glucose ≥7.0 mmol/L, HbA1c ≥6.5% or use of medication to lower blood glucose levels. Dyslipidemia was defined as (any of all) low-density lipoprotein cholesterol ≥3.63 mmol/L, triglyceride ≥1.70 mmol/L, high-density lipoprotein cholesterol <1.04 mmol/L or use of medication to treat serum lipid abnormalities. A clinical diagnosis of sleep apnoea syndrome (SAS) was also collected by the questionnaire.

### Other possible confounding parameters

We evaluated oxygen saturation (SpO2) during sleep and physical activity in limited numbers of participants as possible confounding parameters.

### SpO2 during sleep

In 587 participants, SpO2 during sleep was continuously measured for one night using a pulse oximeter (PULSOX-Me300, Nihon Kohden, Co., Tokyo, Japan), with a finger-type probe that measured light absorption at 665 and 880 nm (LM-5C, Nihon Kohden). Mean SpO2 during sleep and 3% oxygen desaturation index (3% ODI), namely the number of SpO2 drops greater than 3% per hour, were calculated with dedicated Windows software (DS-Me, Nihon Kohden), using data stored in the equipment, and bed and wake-up times obtained by the questionnaire^18^.

### Physical activity

In 566 participants, whole day physical activity was measured with an accelerometer (Lifecorder EX device, Suzuken, Co., Nagoya, Japan) for consecutive 2 to 7 day periods. Data were retrieved with manufacturer-provided physical activity analysis software, and mean daily motor activity, expressed as calorie expenditure by physical activity, and number of steps were used as indices for physical activity^19^.

### Statistical analyses

Differences in numeric variables were assessed using analysis of variance. Frequency differences were assessed with χ^2^ tests. Covariate adjustment was performed by linear regression analysis. Covariate-adjusted odds ratios for the presence of MCI were evaluated with logistic regression analysis. All statistical analyses were performed with commercially available statistical software (JMP version 10.0.2; SAS Institute Inc., Cary, NC, USA), and p < 0.05 was considered statistically significant.

## Results

### Clinical characteristics of MCI

**Figure 1:**
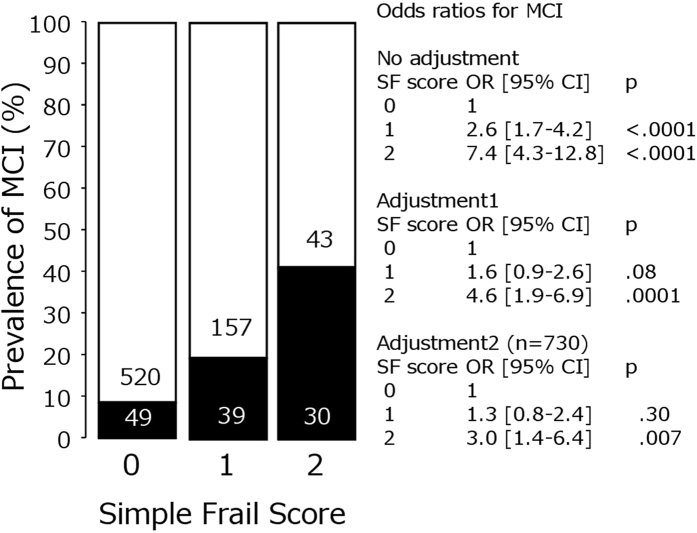
Simple frailty score and the presence of mild cognitive impairment. The closed column indicates the number of participants with mild cognitive impairment (MCI), and the open column indicates those without MCI. The number in the column represents the number of participants. The odds ratio on the right side indicates the odds ratio of a simple frailty (SF) score of 1 and a SF score of 2 to an SF score of 0 for the presence of MCI. Adjustment 1: adjusted for age and sex. Adjustment 2: adjusted for age, sex, body mass index, mean blood pressure, triglyceride, total cholesterol, high-density lipoprotein cholesterol, glucose, insulin, use of antihypertensive drugs, antidyslipidemic drugs, diabetic drugs, current smoking, physical activity and the presence of silent cerebral infarctions and white matter hyperintensity. Adjustment was performed by logistic regression analyses for the presence of MCI. OR, odds ratio; CI, confidence interval.

The clinical characteristics of the study population by the presence/absence of MCI are summarised in Table 1. MCI was observed in 14.1% of participants, and was more prominent in men. Participants with MCI were significantly older than those without MCI. PP, especially radial PP2, was significantly higher in participants with MCI, whereas other parameters showed no significant differences after adjustment for age and sex. Participants with MCI had significantly higher SF scores and SF strict scores (Fig. 1, Supplementary Fig. 1). Odds ratios for an SF score of 2 to a score of 0 for MCI were significantly higher, even after adjusting for possible confounding parameters. For SF strict scores, a score of 1 was significantly associated with MCI. Associations with each component of SF score and SF strict score are shown in Supplementary Figs 2 and 3, indicating that short OLS time accounted for the presence of MCI.

With ROC curve obtained definition, sex and age adjusted odds ratios for the presence of MCI were: SF score 1 to 0, 1.54 (0.88–2.75, p = 0.13) and SF score 2 to 0, 3.39 (1.81–6.42, p = 0.0001).

### MCI and central BP

**Figure 2:**
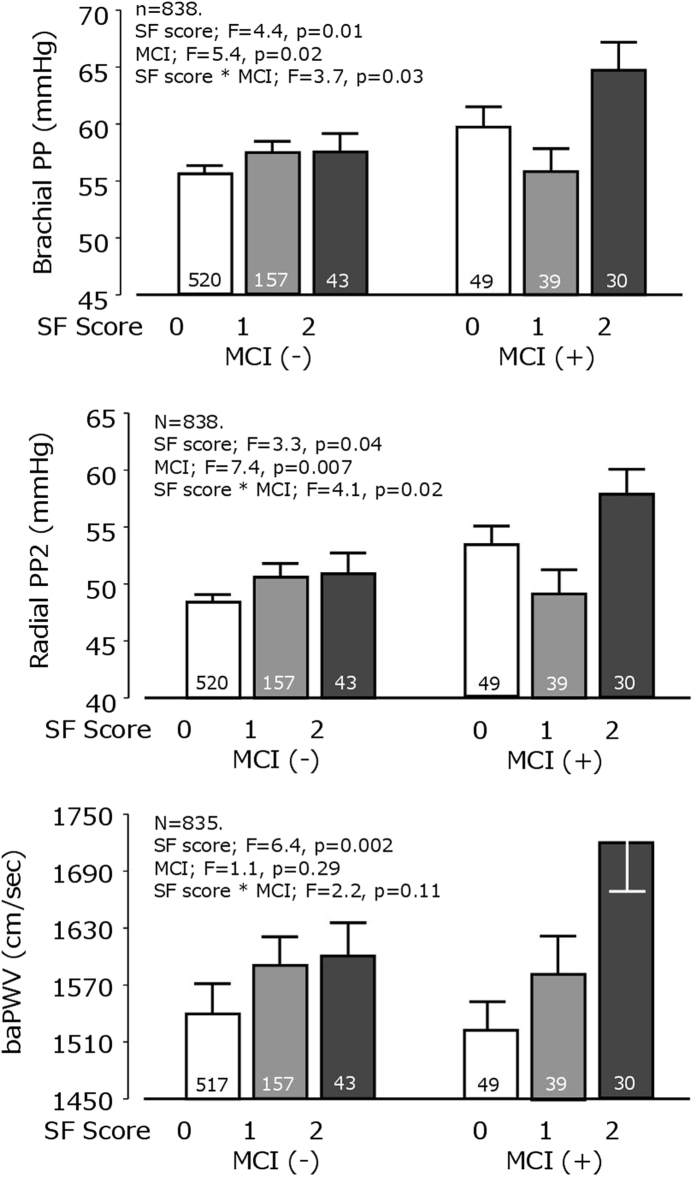
Relationship between simple frailty (SF) score, mild cognitive impairment (MCI) and brachial pulse pressure 2 (PP2), brachial pulse pressure (PP) and brachial-ankle pulse wave velocity (baPWV). The number in the column represents the number of participants. Adjustment was made for age, sex and mean blood pressure. Adjustment was performed using linear regression analysis with interactions between SF score and MCI presence.

Brachial PP and radial PP2 were significantly higher in participants with MCI. Interactions between SF score and MCI status on PP and PP2 were further evaluated (Fig. 2). Both SF score and MCI were significantly associated with PP and radial PP2. However, baPWV (another stiffness index) was only associated with SF score. Similar findings were observed for SF strict score (Supplementary Fig. 4).

### MCI, SF score and SVD

**Figure 3:**
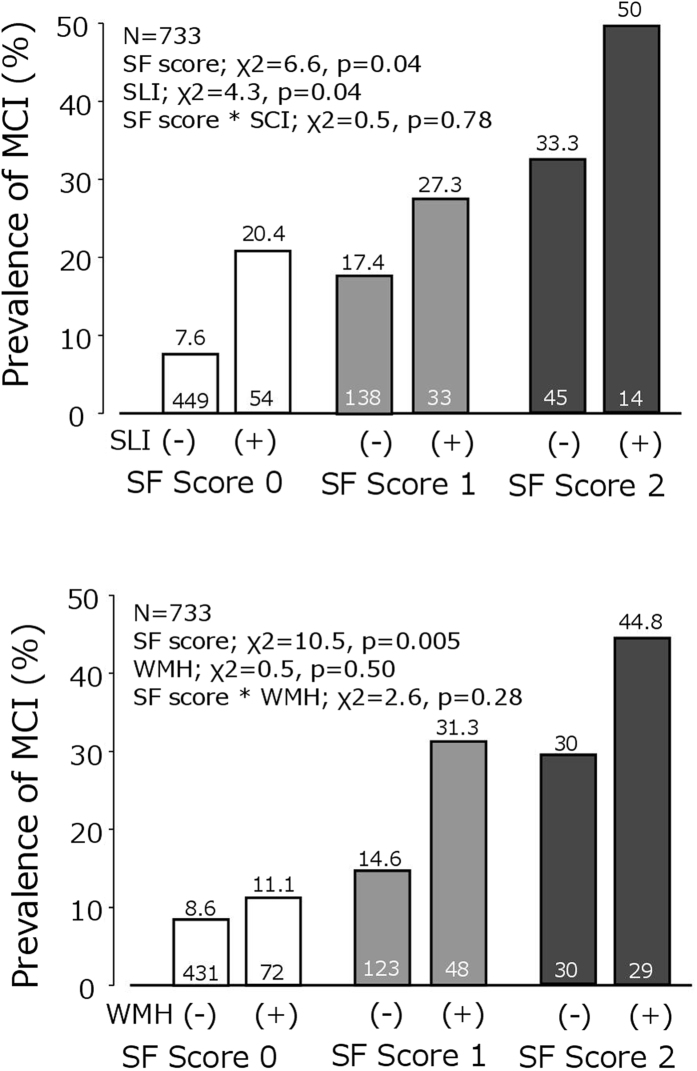
Relationship between simple frailty (SF) score, presence of silent lacunar infarction (SLI), white matter hyperintensity (WMH) and mild cognitive impairment (MCI). The number in the column represents the number of participants. The number above the column indicates the prevalence (%) of MCI. Adjustment for age and sex was performed with logistic regression analyses with interactions between SF score and the presence of SLI or WMH.

In a separate analysis, the presence of SLI was significantly associated with MCI after adjusting for sex and age, whereas WMH was not (Table 1). We further evaluated the possible interaction between SVD parameters and SF score for the presence of MCI (Fig. 3). Even after adjusting for age and sex, SF score and SLI were independently associated with the presence of MCI. However, inclusion of SF strict score eliminated the statistical significance of WMH (Supplementary Fig. 5).

To determine possible independent predictors for the presence of MCI, we performed stepwise logistic regression analyses in several models (Table 2). In the total population, a SF score of 2 and high PP2 were independently associated with MCI in addition to age and male sex. Similar findings were obtained for SF strict score (Supplementary Table 1). Further, analyses including MRI parameters, baPWV and pulmonary functions showed that a SF score of 2, SF strict scores of 1 and 2, radial PP2 and SLI were independent predictors for the presence of MCI (Table 3, Supplementary Table 2).

### Postural instability and MCI

As short OLS time was associated with MCI, the relationship between postural instability and MCI was further investigated using the path-length of centre of gravity as an index for postural instability. The path-length of centre of gravity was significantly higher in participants with MCI and those with WMH (Supplementary Fig. 6). Further, the interaction between postural instability and SVD was investigated. Postural instability was significantly associated with the presence of MCI, in addition to SLI (Supplementary Fig. 7).

## Discussion

We demonstrated that an office-based easily obtainable SF score predicted the presence of MCI in an apparently healthy, independent population. We also demonstrated that central PP elevation and SLI were independently associated with MCI. These findings indicate that physical frailty underlies MCI, in addition to subclinical brain damage and central hemodynamic alteration.

The close association between cognitive impairment and frailty has been previously studied^20^,^21^. We recently reported that an easily measured simple physical frailty score can show systemic frailty (including cognitive function) as well as advanced end organ damage^10^. In the present study, we showed that the SF score (combination of hand grip strength and OLS time) is useful to predict the presence of amnestic MCI. Further analysis revealed that low hand grip strength alone was not associated with MCI, although it was significantly associated with cognitive test score in both men and women (data not shown). SF strict score based on the Asian definition of sarcopenia (grip strength of 26/19 kg) significantly predicted the presence of MCI. Interestingly, postural instability also significantly predicted the presence of MCI. However, the association between OLS time and path-length of centre of gravity was modest (r = −0.39, p < .0001); indicating short OLS time was not only a reflection of postural instability.

Several studies have reported an association between low hand grip strength and slow processing speed^22^ and dementia^23^,^24^. In our study, short OLS time alone was significantly associated with MCI. However, short OLS time combined with low hand grip strength further increased the odds ratio for the presence of MCI (Supplementary Figs 2 and 3), indicating a synergistic effect between hand grip strength and OLS time as an index for frailty.

Although our original SF score predicted the presence of end-organ damage^10^, we also evaluated this association using a more strict definition of low grip strength (Asian definition of sarcopenia). Interestingly, this SF strict score was significantly and independently associated with MCI (even a SF strict score of 1) indicating that the Asian definition may be most suitable for such a prediction. We also evaluated the prediction of MCI using a definition of low grip strength and low OLS time from the ROC curve for each parameter. With this definition, sex and age adjusted odds ratios for the presence of MCI were similar to our original SF score. These findings also increased the complexity of determining cut-off values for the SF score.

Cardiovascular risk factors have been shown to be involved in the development of MCI^25^,^26^. However, we found that only BP parameters were related to amnestic MCI after adjustment for age and sex. As there was a significant difference in age and sex between participants with and without MCI, adjustment for age might have obscured possible associations. In our raw data, the prevalence of hypertension and diabetes was significantly higher in participants with MCI. MCI was also associated with higher PP, especially radial PP2 (a surrogate index for aortic PP). We demonstrated that low hand grip strength was associated with high central BP via arterial stiffness^12^. Therefore, we speculated that SF score may underlie the association between high central PP and MCI. However, stepwise logistic regression analysis showed that radial PP2 and SF score were independently associated with the presence of MCI.

The advantage of the SF score is that it is easily measured in the clinician’s office during patient visits, whereas gait speed measurement requires a straight line distance of 5–6 meters to walk. SF score may therefore be a useful tool to support case-findings by physicians and health professionals in their daily practice. In addition, SF score does not depend on assessment of cognitive function, which may make patients uncomfortable, especially those without subjective symptoms.

Identification of MCI is clinically useful for early intervention in the development of dementia, as well as for careful management of BP. To date, there has been no data available about how BP should be controlled in patients with hypertension who also have cognitive impairment or dementia^27^. A recent sub-analysis of older adults in the Systolic Blood Pressure Intervention Trial (SPRINT) study indicated that irrespective of levels of frailty, strict management of SBP (as low as 120 mmHg) in older adults aged over 75 years significantly reduced cardiovascular events and total mortality^28^. In contrast, Mossello *et al*.^29^ reported that lowering SBP (<125 mmHg) using antihypertensive drugs increased the progression of cognitive decline in patients with hypertension and MCI or dementia. Several other studies have also reported a possible negative effect of extensive BP reduction on cognitive function^30^,^31^. These findings indicate it is important to detect cognitive impairment in older adult patients with hypertension to determine appropriate levels for BP control.

Associations between respiratory disorders, including SAS and chronic obstructive pulmonary disease, and MCI have been demonstrated^32^,^33^. In the present study, the prevalence of SAS and nocturnal decline of SpO2 did not significantly differ after correction for age and sex (Table 1). Pulmonary function, especially VC and %VC, showed a significant declined in participants with MCI. However, in the logistic regression analyses, pulmonary functions were not independently associated with MCI. As a close association between sarcopenia and pulmonary dysfunction has been demonstrated^10^, SF score could include pulmonary dysfunction.

There are several limitations to consider in interpreting our results. The cross-sectional nature of the study design does not show causality between higher SF scores and MCI. Since we quantified physical activity by accelerometer in only limited number of subjects, we used physical activity score in stepwise logistic analyses. Although physical activity scores were significantly related to both mean daily energy expenditure by physical activity and steps measured by accelerometer (data not shown), physical activity score was too simple to quantify the physical activity. Given the relatively healthy study population, our findings may not be applicable for more frail patients, including those residing in nursing homes. Replication with a large sample of heterogeneous subjects in a longitudinal observation would be necessary to confirm our findings. Sub-analysis of cognitive function in SPRINT study may provide further insight for the management of BP in patients who are hypertensive and have cognitive impairment.

In summary, SF score is a significant predictor of the presence of MCI, independent of central PP and subclinical brain damage, which are also associated with SF score. Our findings further support a close association between physical frailty and cognitive impairment. Our simple, office-based evaluation of frailty may help to identify older adults at high risk for frailty and MCI to guide further intervention.

## Additional Information

**How to cite this article:** Ohara, M. *et al*. Office-based simple frailty score and central blood pressure predict mild cognitive impairment in an apparently healthy Japanese population: J-SHIPP study. *Sci. Rep.* **7**, 46419; doi: 10.1038/srep46419 (2017).

**Publisher's note:** Springer Nature remains neutral with regard to jurisdictional claims in published maps and institutional affiliations.

## Supplementary Material

Supplementary Information

## Figures and Tables

**Table 1:** Clinical characteristics of the study population by the presence/absence of mild cognitive impairment.

	MCI (−)	MCI (+)	P
N	720	118	
Male, n (%)	255 (35)	64 (54)	<0.0001
Age, years	64.0 (9.6)	71.9 (7.9)	<0.0001
Body height, cm	159.3 (5.4)	158.1 (5.4)	0.03
Body weight, kg	59.9 (8.6)	59.3 (8.6)	0.55
Body mass index, kg/m^2^	23.5 (3.2)	23.6 (3.2)	0.72
Systolic blood pressure, mmHg	132.0 (18.6)	134.4 (18.5)	0.19
Diastolic blood pressure, mmHg	75.8 (11.3)	74.7 (11.1)	0.37
Pulse pressure, mmHg	56.2 (13.4)	59.7 (13.3)	0.01
Radial Systolic blood pressure 2, mmHg	124.4 (19.3)	127.4 (19.2)	0.13
Radial pulse pressure2, mmHg	48.7 (14.0)	52.6 (13.9)	0.005
Heart rate, beats/min	65.7 (9.7)	66.3 (9.7)	0.52
Triglyceride, mg/dl	110.6 (61.2)	111.5 (60.6)	0.89
Total cholesterol, mg/dl	215.7 (38.1)	213.6 (37.7)	0.58
HDL cholesterol, mg/dl	64.5 (16.9)	64.7 (16.6)	0.92
Fasting glucose, mg/dl	103.8 (19.6)	105.6 (19.3)	0.34
Insulin, pg/ml	6.3 (4.6)	5.7 (4.5)	0.18
Antihypertensive drugs use, n (%)	217 (30)	46 (39)	0.39
Antidyslipidemia drugs use, n (%)	194 (27)	34 (29)	0.48
Antidiabetic drugs use, n (%)	37 (5)	14 (12)	0.09
Hypertension, n (%)	342 (48)	76 (64)	0.65
Dyslipidemia, n (%)	485 (67)	77 (65)	0.67
Diabetes, n (%)	54 (9)	21 (19)	0.21
Sleep apnoea, n (%)	36 (5)	4 (4)	0.43
Smoking status	45/187/488	9/37/72	0.33
Physical activity status	106/384/185/45	35/60/20/3	0.52
baPWV, cm/sec (n = 835)	1564 (295)	1592 (292)	0.34
MRI findings (n = 733)	N = 632	N = 101	
Silent lacunar infarct, n (%)	74 (12)	27 (27)	0.02
PVH >= 2, n (%)	99 (16)	33 (33)	0.30
DSWMH >= 3, n (%)	48 (8)	20 (20)	0.10
WMH, n (%)	113 (18)	36 (36)	0.29
Saturation monitor during sleep (n = 587)	N = 511	N = 76	
Mean SpO2	95.9 (1.3)	96.1 (1.3)	0.37
3% ODI, events/hr	8.8 (7.8)	8.8 (7.1)	0.56
Dip bottom SpO2	91.4 (2.1)	91.5 (2.1)	0.60
Respiratory function (n = 826)	N = 709	N = 117	
Vital capacity, L	2.76 (0.5)	2.64 (0.5)	0.02
%VC, %	101.0 (17.5)	96.4 (18.2)	0.01
Forced VC, L	2.50 (0.5)	2.42 (0.5)	0.16
FEV1.0, L	2.15 (0.4)	2.08 (0.5)	0.13
FEV1.0%, %	90.9 (8.4)	90.8 (8.3)	0.94
Active meter (n = 566)	N = 495	N = 71	
Mean motor activity, kcal/day	175.8 (86.2)	180.8 (10.527)	0.66
Steps, number/day	7169 (2857)	7131 (2938)	0.92

Values are mean (standard deviation) or number (%). MCI, mild cognitive impairment; HDL, high density lipoprotein; baPWV, brachial-ankle pulse wave velocity; MRI, magnetic resonance imaging; PVH, periventricular hyperintensity; DSWMH, deep subcortical white matter hyperintensity; WMH, white matter hyperintensity; SpO2, oxygen saturation; ODI, oxygen desaturation index; VC, vital capacity; FEV 1.0, forced expiratory volume in one second. All values except for age, sex and MRI parameters were adjusted for age and sex.

**Table 2:** Stepwise logistic regression analysis for the presence of mild cognitive impairment in the total study population.

N = 838	Odds ratio	95% CI	P
Sex, male = 1	2.66	1.73–4.14	0.0004
Age, 10 years	2.03	1.49–2.80	<0.0001
Body mass index			
Mean blood pressure, mmHg			
Brachial pulse pressure, 10 mmHg			
Radial pulse pressure 2, 10 mmHg	1.19	1.03–1.36	0.018
Triglyceride, mg/dl			
Total cholesterol, mg/dl			
HDL cholesterol, mg/dl			
Fasting glucose, mg/dl			
IRI, μU/ml			
Antihypertensive drugs use			
Antidyslipidemia drugs use			
Antidiabetic drugs use			
Current smoking, yes = 1			
Physical activity^1)^			
Simple frail score 1 vs. 0	1.52	0.92–2.51	0.10
Simple frail score 2 vs. 0	3.58	1.86–6.86	0.0001

HDL, high-density lipoprotein; IRI, immune-reactive insulin; CI, confidence interval. 1) Physical activity (every day = 1, sometimes = 2, not often = 3, never = 4). Blank columns indicate parameters not entered in the equation.

**Table 3:** Stepwise logistic regression analysis for the presence of mild cognitive impairment, including brain lesions.

N = 733	Odds ratio	95% CI	P
Sex, male = 1	2.34	1.48–3.74	0.003
Age, 10 years	1.88	1.35–2.65	0.0001
Body mass index			
Mean blood pressure, mmHg			
Brachial pulse pressure, 10 mmHg			
Radial pulse pressure 2, 10 mmHg	1.18	1.02–1.38	0.029
Triglyceride, mg/dl			
Total cholesterol, mg/dl			
HDL cholesterol, mg/dl			
Fasting glucose, mg/dl			
IRI, μU/ml			
Antihypertensive drugs use			
Antidyslipidemia drugs use			
Antidiabetic drugs use			
Current smoking, yes = 1			
Physical activity^1)^			
baPWV, 100 cm/sec			
Silent lacunar infarct, yes = 1	1.83	1.04–3.14	0.035
White matter hyperintensity, yes = 1			
%VC, %			
FEV1.0%, %			
Simple frail score 1 vs. 0	1.37	0.80–2.34	0.25
Simple frail score 2 vs. 0	2.99	1.46–6.04	0.003

HDL, high-density lipoprotein; IRI, immune-reactive insulin; CI, confidence interval; VC, vital capacity; FEV1.0%, forced expiratory volume % in one second. Physical activity (every day = 1, sometimes = 2, not often = , never = 4). Blank columns indicate parameters not entered in the equation.
