# Supplementary material for: Office-based simple frailty score and central blood pressure predict mild cognitive impairment in an apparently healthy Japanese population: J-SHIPP study
Source: Sci Rep. 2017 Apr 13;7:46419. doi: 10.1038/srep46419 (PMC5390291; doi:10.1038/srep46419)
Supplement: Supplementary Information [file srep46419-s1.pdf]

Supplementary Table 1.

Office-based simple frailty score and central blood pressure predict mild cognitive impairment in an apparently healthy Japanese population: J-SHIPP study by Maya Ohara, Katsuhiko Kohara, Yoko Okada, Masayuki Ochi, Tokihisa Nagai, Yasumasa Ohyagi, Yasuharu Tabara, Michiya Igase.

Stepwise logistic regression analysis for the presence of mild cognitive impairment in the total study population

N=838	Odds ratio	95% CI	P
Sex, male=1	2.92	1.88-4.60	<.0001
Age, 10 years	2.05	1.51-2.80	<.0001
Body mass index			
Mean blood pressure, mmHg			
Brachial pulse pressure, 10 mmHg			
Radial pulse pressure 2, 10 mmHg	1.18	1.03-1.36	.019
Triglyceride, mg/dl			
Total cholesterol, mg/dl			
HDL cholesterol, mg/dl			
Fasting glucose, mg/dl			
IRI, μU/ml			
Antihypertensive drugs use			
Antidyslipidemia drugs use			
Antidiabetic drugs use			
Current smoking, yes=1			
Physical activity[1)]			
Simple frail strict score 1 vs. 0	2.34	1.43-3.82	.0007
Simple frail strict score 2 vs. 0	4.58	1.99-10.3	.0004

HDL, high-density lipoprotein; IRI, immune-reactive insulin; CI, confidence interval.

1) Physical activity (every day=1, sometimes=2, not often=3, never=4).

Blank columns indicate parameters not entered in the equation.

Supplementary Table 2.

Office-based simple frailty score and central blood pressure predict mild cognitive impairment in an apparently healthy Japanese population: J-SHIPP study by Maya Ohara, Katsuhiko Kohara, Yoko Okada, Masayuki Ochi, Tokihisa Nagai, Yasumasa Ohyagi, Yasuharu Tabara, Michiya Igase.

Stepwise logistic regression analysis for the presence of mild cognitive impairment, including brain lesions

N=733	Odds ratio	95% CI	P
Sex, male=1	2.66	1.65-4.34	<.0001
Age, 10 years	1.88	1.36-2.64	<.0001
Body mass index			
Mean blood pressure, mmHg			
Brachial pulse pressure, 10 mmHg			
Radial pulse pressure 2, 10 mmHg	1.18	1.01-1.37	.035
Triglyceride, mg/dl			
Total cholesterol, mg/dl			
HDL cholesterol, mg/dl			
Fasting glucose, mg/dl			
IRI, μU/ml			
Antihypertensive drugs use			
Antidyslipidemia drugs use			
Antidiabetic drugs use			
Current smoking, yes=1			
Physical activity[1)]			
baPWV, 100 cm/sec			
Silent lacunar infarct, yes=1	1.87	1.07-3.22	.03
White matter hyperintensity, yes=1			
%VC, %			
FEV1.0 %, %			
Simple frail strict score 1 vs. 0	2.03	1.19-3.43	.01
Simple frail strict score 2 vs. 0	4.84	1.94-11.7	.001

HDL, high-density lipoprotein; IRI, immune-reactive insulin; CI, confidence interval.

1) Physical activity (every day=1, sometimes=2, not often=, never=4). Blank columns indicate parameters not entered in the equation.

Supplementary Figure 1.

Office-based simple frailty score and central blood pressure predict mild cognitive impairment in an apparently healthy Japanese population: J-SHIPP study by Maya Ohara, Katsuhiko Kohara, Yoko Okada, Masayuki Ochi, Tokihisa Nagai, Yasumasa Ohyagi, Yasuharu Tabara, Michiya Igase.

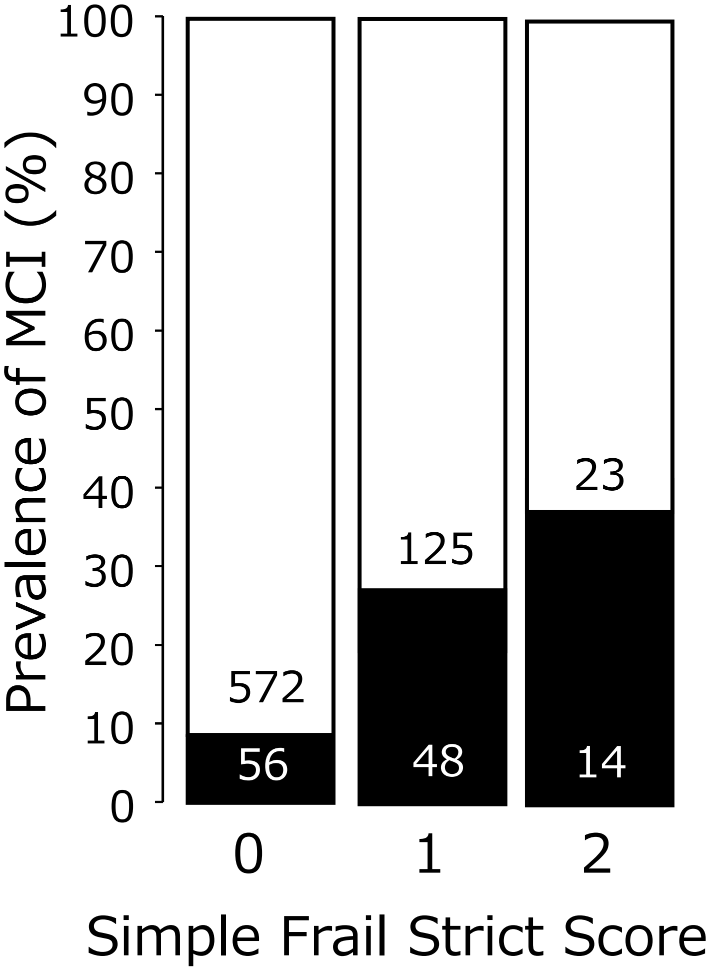

Odds ratios for MCI

No adjustment

SF score	OR [95% CI]	p
0	1	
1	3.9 [2.5-6.0]	<.0001
2	7.4 [3.0-12.6]	<.0001

Adjustment1

SF score	OR [95% CI]	p
0	1	
1	2.3 [1.4-3.7]	.001
2	4.9 [2.1-11.0]	.0003

Adjustment2

SF score	OR [95% CI]	p
0	1	
1	2.1 [1.2-3.6]	.01
2	5.1 [1.9-13.2]	.001

Simple frailty strict score and presence of mild cognitive impairment. The closed column indicates the number of participants with mild cognitive impairment (MCI) and the open column indicates those without MCI. The number in the column represents the number of participants. The odds ratio on the right-hand side indicates the odds ratio of a simple frailty (SF) strict score of 1 and a SF strict score of 2 to a SF strict score of 0 for the presence of MCI. Adjustment 1: adjusted for age and sex. Adjustment 2: adjusted for age, sex, body mass index, mean blood pressure, triglyceride, total cholesterol, high-density lipoprotein cholesterol, glucose, insulin, use of antihypertensive drugs, antidyslipidemic drugs, diabetic drugs, current smoking, physical activity, and the presence of silent cerebral infarctions and white matter hyperintensity. Adjustment was performed by logistic regression analyses for the presence of MCI. OR, odds ratio; CI, confidence interval.

Supplementary Figure 2.

Office-based simple frailty score and central blood pressure predict mild cognitive impairment in an apparently healthy Japanese population: J-SHIPP study by Maya Ohara, Katsuhiko Kohara, Yoko Okada, Masayuki Ochi, Tokihisa Nagai, Yasumasa Ohyagi, Yasuharu Tabara, Michiya Igase.

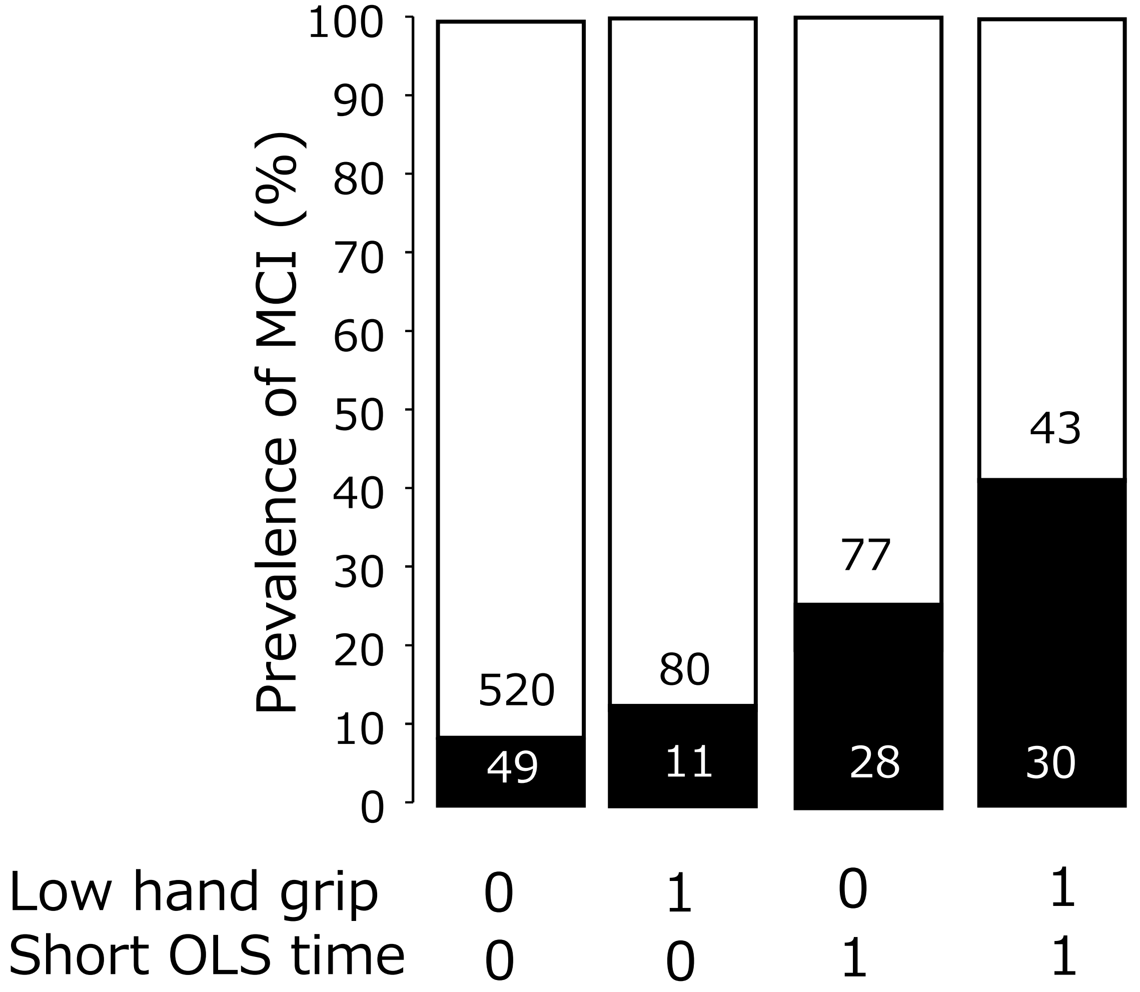

Odds ratios for MCI

	No adjustment OR [95% CI]	p	adjustment OR [95% CI]	p
Low handgrip only	1.5 [0.7-2.8]	.30	0.9 [0.4-1.8]	.79
Short OLS time only	3.9 [2.3-6.5]	<.0001	2.2 [1.2-3.9]	.01
Both	7.4 [4.3-12.8]	<.0001	4.3 [2.1-8.5]	<.001

Relationship of low hand grip strength and short one-leg standing time to the prevalence of mild cognitive impairment. Components of the simple frailty score (low hand grip strength and short one-leg standing time) and their association with mild cognitive impairment (MCI) are shown. The number in the column represents the number of participants. OLS, one-leg standing time; OR, odds ratio; CI, confidence interval. Adjusted for age and sex.

Supplementary Figure 3.

Office-based simple frailty score and central blood pressure predict mild cognitive impairment in an apparently healthy Japanese population: J-SHIPP study by Maya Ohara, Katsuhiko Kohara, Yoko Okada, Masayuki Ochi, Tokihisa Nagai, Yasumasa Ohyagi, Yasuharu Tabara, Michiya Igase.

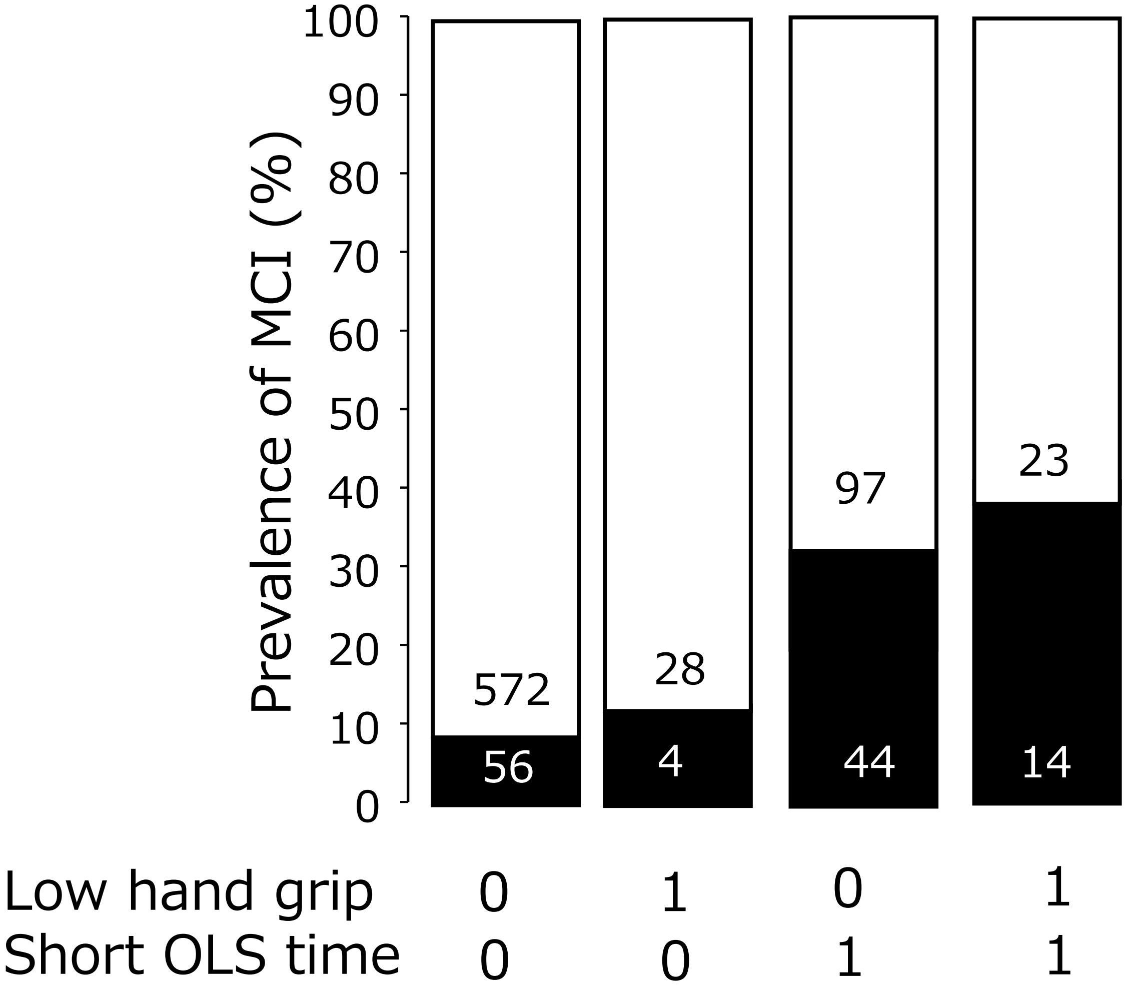

Odds ratios for MCI

	No adjustment OR [95% CI]	p	adjustment OR [95% CI]	p
Low handgrip only	1.5 [0.4-3.9]	.51	1.8 [0.5-5.6]	.34
Short OLS time only	4.6 [2.9-7.3]	<.0001	2.1 [1.2-3.7]	.01
Both	6.2 [3.0-12.6]	<.0001	5.1 [1.9-13.2]	.001

Relationship of low hand grip strength and short one-leg standing time to the prevalence of mild cognitive impairment. Components of the simple frailty strict score (low hand grip strength and short one-leg standing time) and their association with mild cognitive impairment (MCI) are shown. The number in the column represents the number of participants. OLS, one-leg standing time; OR, odds ratio; CI, confidence interval. Adjusted for age and sex.

Supplementary Figure 4.

Office-based simple frailty score and central blood pressure predict mild cognitive impairment in an apparently healthy Japanese population: J-SHIPP study by Maya Ohara, Katsuhiko Kohara, Yoko Okada, Masayuki Ochi, Tokihisa Nagai, Yasumasa Ohyagi, Yasuharu Tabara, Michiya Igase.

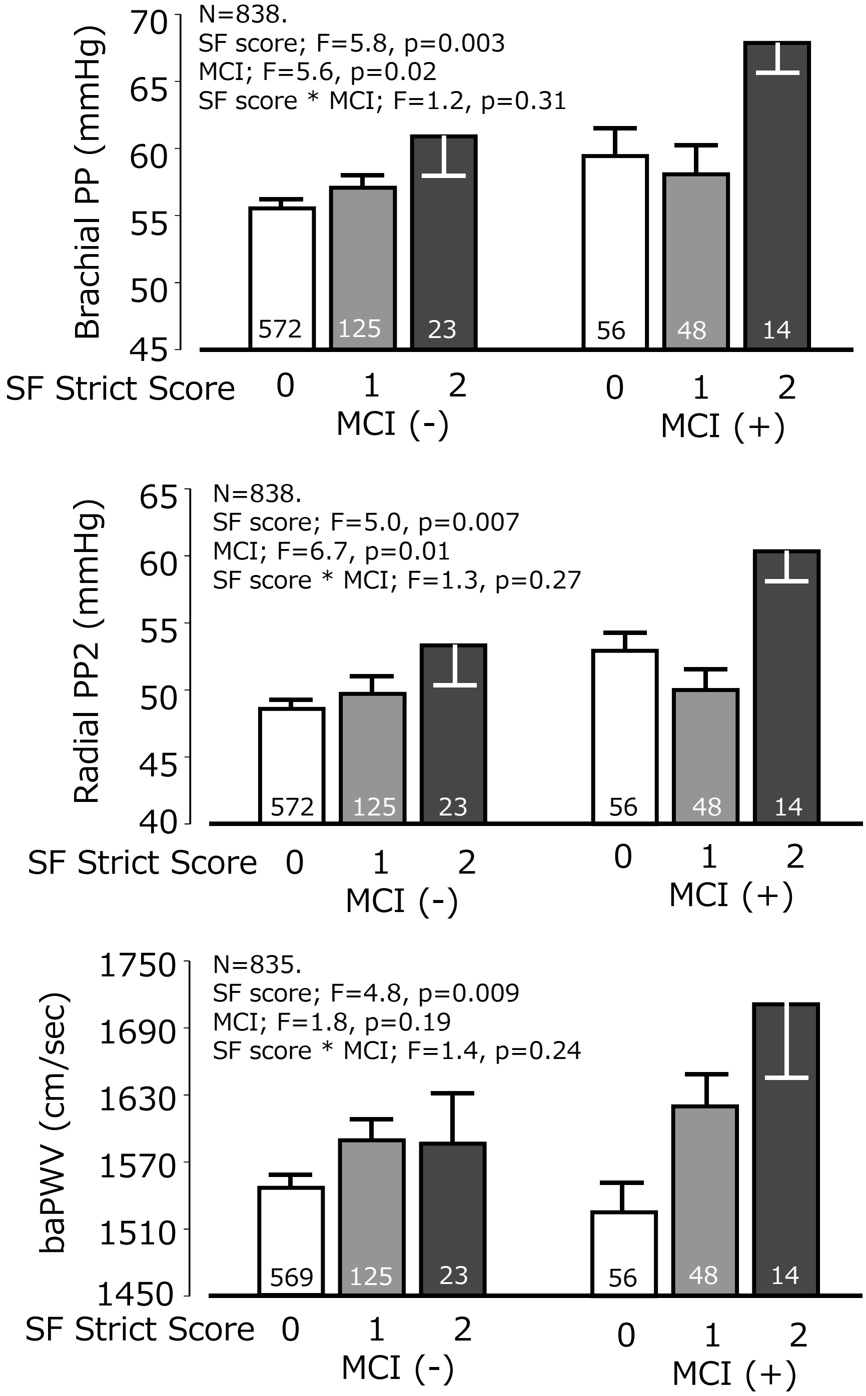

Relationship between simple frailty (SF) strict score, mild cognitive impairment (MCI) and brachial pulse pressure 2 (PP2), brachial pulse pressure (PP) and brachial-ankle pulse wave velocity (baPWV). The number in the column represents the number of participants. Adjustment was made for age, sex and mean blood pressure. Adjustment was performed using linear regression analysis with interactions between SF score and presence of MCI. Values are mean±SEM.

Supplementary Figure 5.

Office-based simple frailty score and central blood pressure predict mild cognitive impairment in an apparently healthy Japanese population: J-SHIPP study by Maya Ohara, Katsuhiko Kohara, Yoko Okada, Masayuki Ochi, Tokihisa Nagai, Yasumasa Ohyagi, Yasuharu Tabara, Michiya Igase.

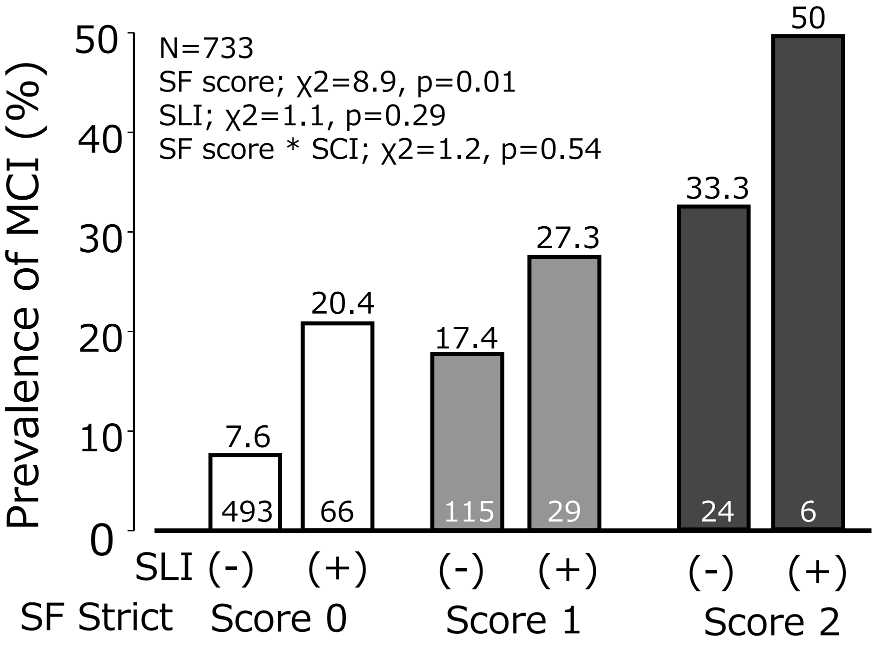

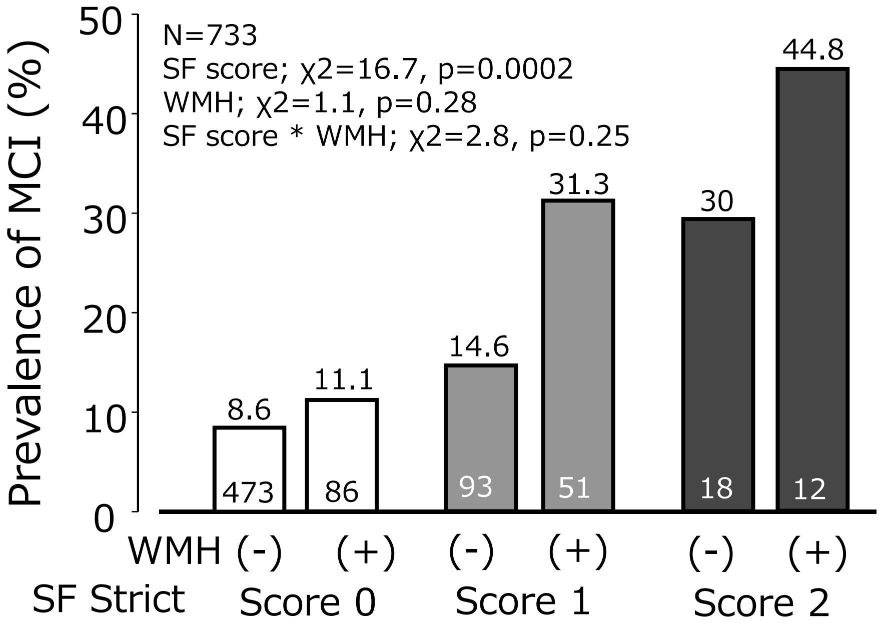

RRelationship between simple frailty (SF) strict score, presence of silent lacunar infarction (SLI), white matter hyperintensity (WMH) and mild cognitive impairment (MCI). The number in the column represents the number of participants. The number above the column indicates the prevalence (%) of MCI. Adjustment for age and sex was performed by logistic regression analyses with interactions between SF score and the presence of SLI or WMH. Values are mean±SEM.

Supplementary Figure 6.

Office-based simple frailty score and central blood pressure predict mild cognitive impairment in an apparently healthy Japanese population: J-SHIPP study by Maya Ohara, Katsuhiko Kohara, Yoko Okada, Masayuki Ochi, Tokihisa Nagai, Yasumasa Ohyagi, Yasuharu Tabara, Michiya Igase.

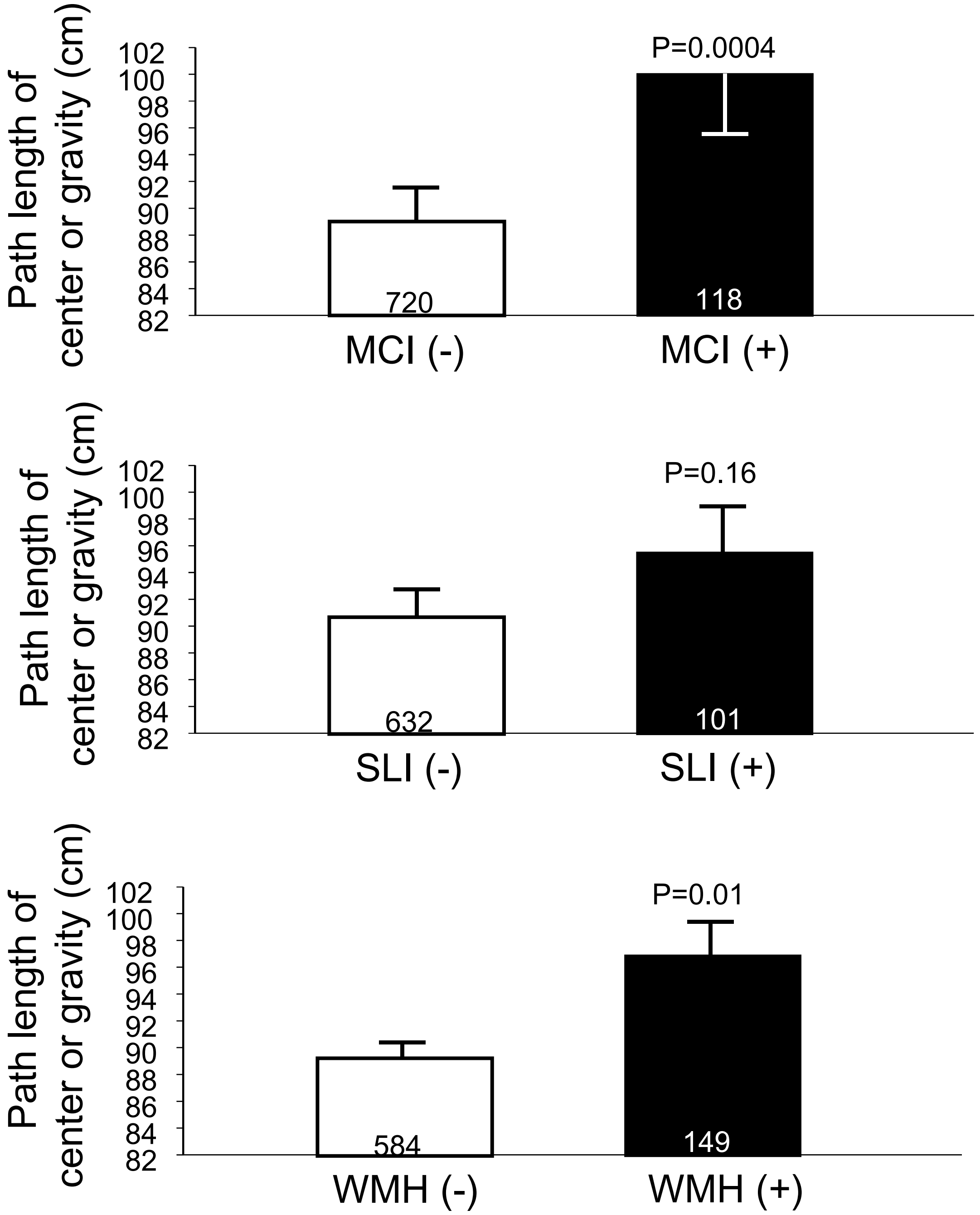

Path length of centre of gravity and mild cognitive impairment (MCI) (top), silent lacunar infarction (SLI) (middle), white matter hyperintensity (WMH) (bottom). The number in the column represents the number of subjects. Adjusted for age and sex. Values are mean±SEM.

Supplementary Figure 7.

Office-based simple frailty score and central blood pressure predict mild cognitive impairment in an apparently healthy Japanese population: J-SHIPP study by Maya Ohara, Katsuhiko Kohara, Yoko Okada, Masayuki Ochi, Tokihisa Nagai, Yasumasa Ohyagi, Yasuharu Tabara, Michiya Igase.

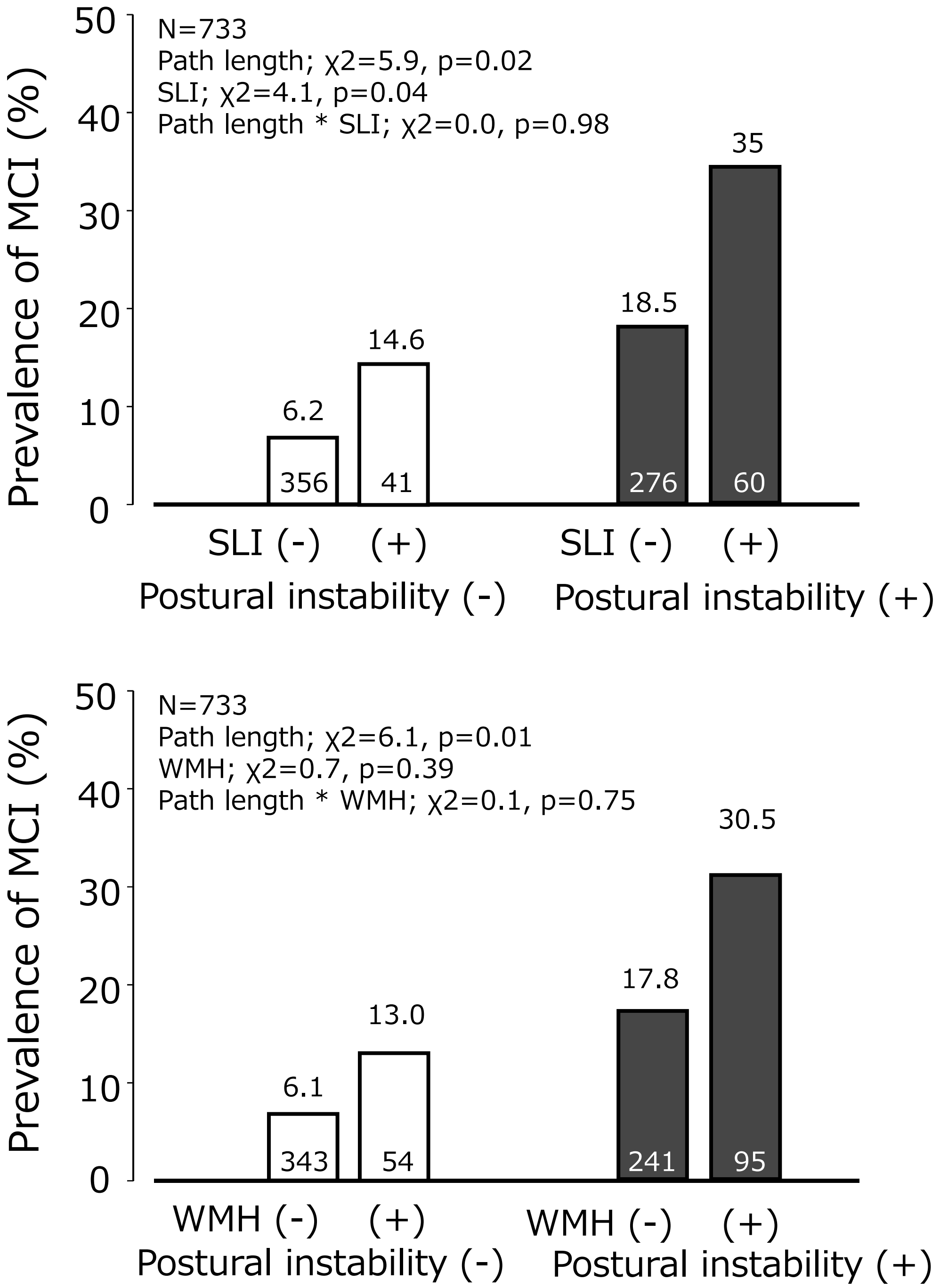

Relationship between postural instability, presence of silent lacunar infarction (SLI), white matter hyperintensity (WMH) and mild cognitive impairment (MCI). The number in the column represents the number of participants. The number above the column indicates the prevalence (%) of MCI. Adjusted for age and sex. Postural instability was defined as a path-length of centre of gravity of 86.92 cm or more. Values are mean±SEM.
